# Stability of SARS‐CoV‐2 as consequence of heating and microwave processing in meat products and bread

**DOI:** 10.1002/fsn3.2481

**Published:** 2021-07-20

**Authors:** Sahar Norouzbeigi, Reza Yekta, Leily Vahid‐Dastjerdi, Hossein Keyvani, Mohammad Mehdi Ranjbar, Mahdi Shadnoush, Mojtaba Yousefi, Nasim Khorshidian, Sara Sohrabvandi, Amir M. Mortazavian

**Affiliations:** ^1^ Student Research Committee Department of Food Science and Technology National Nutrition and Food Technology Research Institute Faculty of Nutrition and Food Technology Shahid Beheshti University of Medical Sciences Tehran Iran; ^2^ Department of Virology School of Medicine Iran University of Medical Sciences Tehran Iran; ^3^ Department of Virology Razi Vaccine and Serum Research Institute, (AREEO) Agricultural Research, Education and Extension Organization Tehran Iran; ^4^ Department of Clinical Nutrition Faculty of Nutrition Sciences and Food Technology National Nutrition and Food Technology Research Institute Shahid Beheshti University of Medical Sciences Tehran Iran; ^5^ Food Safety Research Center (Salt) Semnan University of Medical Sciences Semnan Iran; ^6^ Department of Food Technology Research, Faculty of Nutrition Sciences and Food Technology National Nutrition and Food Technology Research Institute, Shahid Beheshti University of Medical Sciences Tehran Iran; ^7^ Department of Food Science and Technology, Faculty of Nutrition Sciences and Food Technology National Nutrition and Food Technology Research Institute, Shahid Beheshti University of Medical Sciences Tehran Iran

**Keywords:** bread, COVID‐19, food, heat, inactivation, meat, microwave, Virus

## Abstract

The new severe acute respiratory syndrome coronavirus 2 (SARS‐CoV‐2) that was first found in 2019 in Wuhan, China, caused coronavirus disease 2019 (COVID‐19). It then spread worldwide rapidly, causing the 2019–2020 coronavirus pandemic. To date, it has been indicated that various transmission ways might be participated in outbreaks of COVID‐19. Among these, food products, whether raw or processed, might be carriers for SARS‐CoV‐2. Therefore, this study was aimed to evaluate the effect of cooking and microwave process of meat products and bread on the stability of SARS‐CoV‐2. In this regard, sausages and hamburger as meat products and toast bread were inoculated with a viral load of 5.70 log fifty percent tissue culture infective dose (TCID_50_)/mL in order to create a simulated cross‐contamination condition. The results showed that frying of hamburger at 225ºC for about either 6 or 10 min resulted in complete inactivation of SARS‐CoV‐2. Furthermore, a 5‐log decrease in SARS‐CoV‐2 load was observed in sausages as a consequence of cooking process at 78ºC for either 20 or 30 min. Additionally, the effect of microwave oven at power of 630 watt on stability of SARS‐CoV‐2 showed that exposing toast bread for either 30 s or 1 min in this power led to a 5‐log decrease in SARS‐CoV‐2 load in the toast bread.

## Graphical abstract

In this study, stability of SARS‐CoV‐2 toward heating and microwave in meat and bread have been studied for the first time.

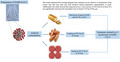

## 1 INTRODUCTION

Coronaviruses are RNA viruses that belong to the subfamily Coronavirinae, family of Coronavirdiae, order of Nidovirales, which can cause different infectious diseases in various hosts such as birds, mice, horses, dogs, cats, cows, pigs, and humans (Li, Liu et al., 2020). Respiratory tract, enteric, hepatic, renal, and central nervous system diseases can be named as various infectious diseases caused by coronavirus in many birds and mammals, such as human (Franks & Galvin, 2014). Furthermore, the common cold, a generally mild acute respiratory illnesses, is caused by coronaviruses as common human pathogens (Won et al., 2020). Prior to December 2019, the outbreaks of two diseases such as severe acute respiratory syndrome (SARS) and Middle Eastern respiratory syndrome (MERS) had been caused by two additional strains of coronaviruses (Shariatifar et al., 2019). At the end of 2019, a new strain of coronavirus that has not been formerly identified in humans was first found as the cause of a cluster of pneumonia cases in Wuhan, Hubei Province, China. After that, the coronavirus disease 2019 (COVID‐19) caused by severe acute respiratory syndrome coronavirus 2 (SARS‐CoV‐2) spread worldwide rapidly (Han et al., 2020; Shariatifar et al., 2019). Due to the significant increase in incidence of disease and mortality, COVID‐19 has caused a great fear among humanity (Shi et al., 2020). Hence, it was considered as a public health emergency of international concern on 30 January 2020, and finally, the World Health Organization (WHO) announced it a pandemic on 11 March 2020 (Director, 2020; Galanakis, 2020).

Coronaviruses that caused MERS and SARS are transmitted usually among animals and humans. Transmission of SARS and MERS to humankind was carried out by Civet cats and camels, respectively; however, bats were a natural source of both (Jalava., 2020; Pressman et al., 2020). Additionally, it has been proposed by some investigations that bats are the probable source of the COVID‐19 outbreaks; however, the intermediate host of COVID‐19 is unknown. It has been suggested that various animals such as pangolins, snakes, turtles, and Pomeranian dogs might be considered as possible intermediate hosts of the COVID‐19 (Dhama et al., 2020; Rodriguez‐Morales et al., 2020). The symptoms of COVID‐19 seem to typically appear after an incubation period of around 5–6 days and up to 14 days in some cases (Li, Guan, et al., 2020). The signs of COVID‐19‐infected people mostly include mild‐to‐moderate respiratory illness and recover without any particular cure. However, COVID‐19 is more likely to be a serious illness in the elderly and people with major medical problems such as cancer cardiovascular disease, chronic respiratory disease, and diabetes (Yari et al., 2020). Headache, dry cough, fever, dyspnea, myalgia or fatigue, and losing smell or taste are the most common symptoms of COVID‐19‐infected people. Furthermore, more signs such as hemoptysis, shortness of breath, and diarrhea rarely appear at the time of hospitalization (Xu et al., 2020). Full awareness of the COVID‐19, the disease, and how it spreads are the best ways to hinder and slow down the rate of COVID‐19 transmission. Direct person‐to‐person transmission has been considered as the main way of transmission of COVID (McIntosh et al., 2020; Yari et al., 2020).

It seems that transmission of COVID‐19 can occur via close‐range contact, especially through droplets that are formed during coughs, sneezes, or talks that releases virus and, therefore, can infect another person (McIntosh et al., 2020). Furthermore, virus detection in saliva, digestive system, feces, and urine indicated that gastrointestinal tract might be considered as another possible transmission route of this virus (Dhama et al., 2020; Pressman et al., 2020; Yekta et al., 2020). In fact, apart from the transmission of COVID‐19 through the respiratory tract, detection of viral RNA in patients' feces implies the possibility of route of COVID‐19 transmission (Yekta et al., 2020; Zhang et al., 2020). Moreover, occurrence of infection can also occur if person contacts and touches virus‐infected surfaces and then touches his or her mouth, nose, and eyes (McIntosh et al., 2020). Therefore, in addition to keep social distance, it is essential to regularly wash and disinfect hands with soap, especially after contact with any person or suspicious things (Biscayart et al., 2020; Jin et al., 2020; Yari et al., 2020).

The stability of virus in different conditions and various surfaces is not completely clear, and it has been indicated that its stability varies from several hours to more than 7 days in inanimate surfaces depending on the type of surface (temperature 22℃ and relative humidity 65%) (Chin et al., 2020; Chin & Poon, 2020). Furthermore, it has been reported that virus is stable for 14 days at 4ºC and with increasing temperature, its stability decreases and in order to completely destroy this virus, temperature of 70ºC for 5 min should be exerted (Chin & Poon, 2020; Kampf et al., 2020).

Due to worldwide spread of COVID‐19 and the high rate of transmission, as well as the uncertainty about the exact mechanism of transmission and its stability in different conditions, it seems that interruption of transmission chain is considered as the best way to prevent outbreak of COVID‐19. It has been indicated that transmission of foodborne viral infections can generally occur through the fecal–oral route (Miranda & Schaffner, 2019). Therefore, food, whether raw or processed, might be contaminated with the SARS‐CoV‐2 and act as a carrier in the transmission of COVID‐19, and thus, virus‐contaminated food can be effective in strengthening the COVID‐19 transmission chain. It has been noted that SARS‐CoV‐2 is active and stable at −20℃ for up to 2 years and refrigerator storage (4–8℃) does not disable coronavirus (Han et al., 2020; Hirneisen et al., 2010). Furthermore, as mentioned before, high‐temperature heating (70℃) for about 5 min is required to inactivate SARS‐CoV‐2 (Chin & Poon, 2020). Also, various food‐processing strategies, including high‐temperature heating, gamma irradiation, ultraviolet light, high hydrostatic pressure, and microwave are utilized to ensure about the safety of food products (Gharibzahedi et al., 2019; Koubaa et al., 2018; Putnik et al., 2020; Stamenković et al., 2019). The latter is always applied on a home scale for heating, thawing, and cooking and can be considered as a possible efficient way for inactivation of SARS‐CoV‐2 since it generates heat. On the other hand, due to the complexity of food matrix and protective effects of food constituent such as protein, carbohydrate, and fat on pathogens, it is important to study the effect of heat processing of food products on destruction and inactivation of SARS‐CoV‐2. Therefore, this study was aimed to evaluate the stability SARS‐CoV‐2 under various heat processing such as pasteurization of sausage, cooking of hamburger as well as bread baking.

## 2 MATERIAL AND METHODS

### 2.1 Culturing of Vero cell and SARS‐CoV‐2

#### 2.1.1 Preparation of Vero cell

A/T175 flask of WHO confluent Vero cell line culture prepared from Reference Keyvan Laboratory was trypsinized, centrifuged, and re‐suspended in 10% fetal bovine serum (Gibco^TM^) and 90% media made of Dulbecco's minimum essential medium (DMEM). Furthermore, cells were subcultured in T25 flasks and incubated at 37˚C and 5% CO_2_ until 80% confluency.

#### 2.1.2 Virus isolation and culturing

Patients who were considered COVID‐19‐positive based on their real‐time PCR analysis (cycle thresholds under (CT) values 15) were utilized in this study. The samples were isolated from the nasopharyngeal cavity using swabs and then transferred to laboratory with viral transportation medium in a biosafety level 3 laboratory. The quantification of virus was carried out after K1 isolate SARS‐CoV‐2 propagations and real‐time PCR. Afterward, in order to food inoculation, fifty percent tissue culture infective dose (TCID_50_) assay was done in 96‐well plate and measurement was carried out by the Reed‐Muench method. K1 isolate with log TCID_50_/mL (virus titer) ~ 7 and CT value 10 was utilized for inoculations into food. All investigations on the virus were performed in a biosafety level 3 laboratory.

#### 2.1.3 Virus concentration and evaluation virus titer (infectivity) by TCID_50_ assay

Polyethylene glycol (PEG) precipitation was applied in order to concentrate the virus and remove cytotoxic agents and/or PCR inhibitors from food samples. In this regard, at first, 10 ml of each sample (9 ml sample +1 ml virus) was mixed with 1.5 ml of the PEG 6,000 stock solutions. The suspensions were then placed in the shaking incubator at 150 rpm for 8 hr at 4℃, and after that, the supernatant was centrifuged at 3,635 g for 50 min. The PEG‐containing supernatants were removed, and the harvested pellet was dissolved in 1 ml phosphate‐buffered saline (PBS) and recentrifuged at 4,173 g for 40 min. The obtained supernatant was passed through a 0.2‐μm sterile membrane filter and transferred into cell culture flasks. Thereupon, the cultured flask was placed in a shaking incubator at 37℃ with soft agitation. Eventually, after removing upper phase, the medium culture with low amount of PBS (%2) was added. Cytopathic effect was examined for all the cultured flasks every 24 hr, and after six days, TCID_50_/mL was measured in three replicates using 96‐well plates for each food.

#### 2.1.4 Real‐time PCR

**FIGURE 1:**
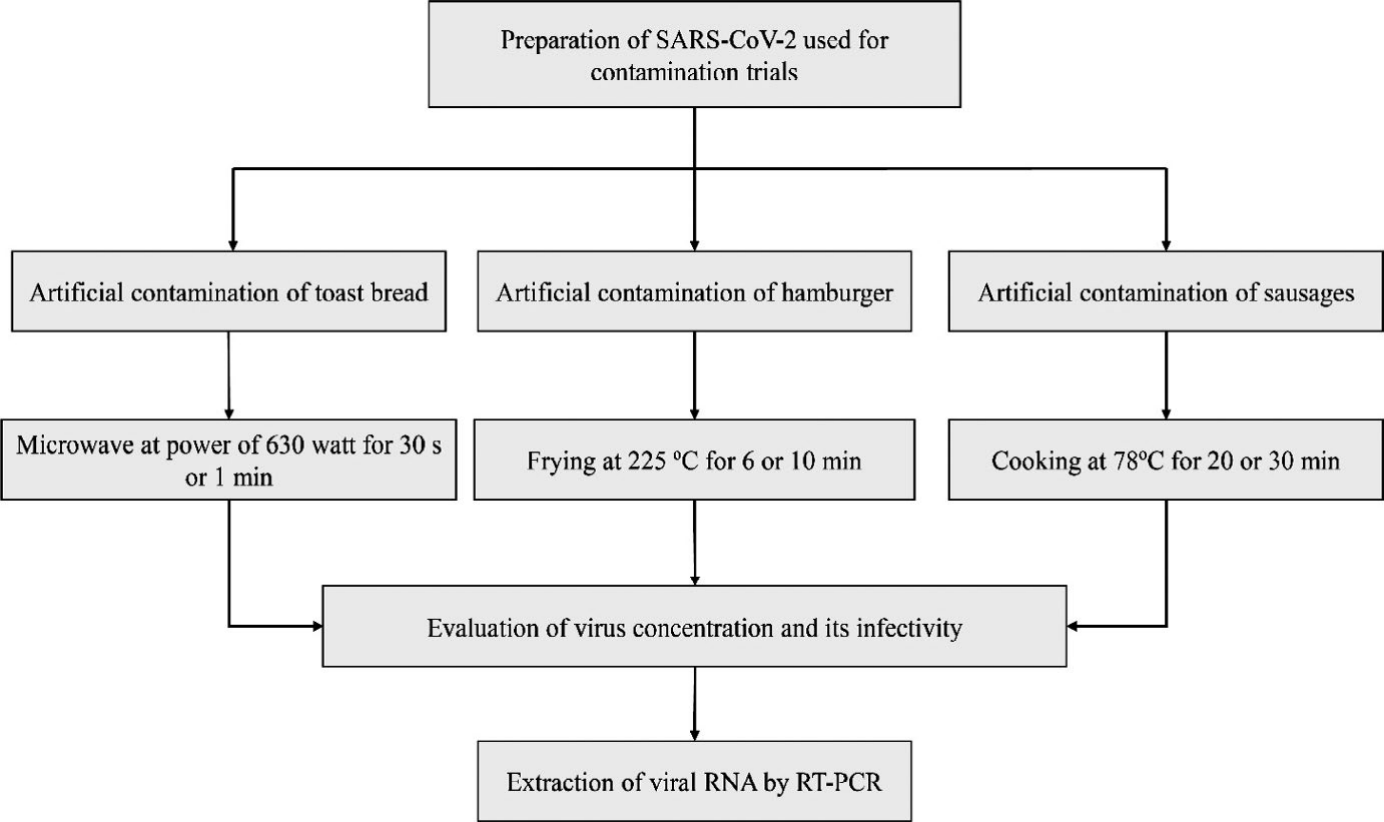
Study design of present work for one replication

All real‐time RT‐PCRs were carried out in the Rotor‐Gene‐Q 6,000 thermocycler (Corbett, Australia) according to the recommended protocol. Briefly, according to Tab ibzadeh et al., (2020), LightMix SarbecoV E‐gene plus EAV control (Roche, Germany) was utilized by using 10 μl extracted RNA or blank, 0.1 μl RT enzyme, 4 μl Roche MasterMix, 0.5 μl primer‐probe mix, and 5.4 μl deionized RNase‐ and DNase‐free water. The thermal cycling conditions were as follows: 3 s at 55˚C and 30 s at 95˚C (1 cycle); 3 s at 95˚C and 12 s at 60˚C (45 cycles); and 10 s at 40˚C (1 cycle) (Tab ibzadeh et al., 2020). The study steps are shown in Figure 1.

### 2.2 Artificial contamination of meat products

In this study, the effect of heating processes (frying and cooking) on inactivation of SARS‐CoV‐2 in artificially contaminated hamburger and sausages were studied. In this regard, 9 g of hamburger was seeded with 1 ml of SARS‐CoV‐2 at a final concentration of ~6 log TCID_50_/g. The contaminated hamburger was fried at 225℃ for 3 and 5 min. Furthermore, two kinds of raw sausage (40 and 80% meat) were seeded with 1 ml of SARS‐CoV‐2 at a final concentration of ~6 log TCID_50_/g. The contaminated sausages were placed in oven in order to cook at 78℃ for 20 and 30 min.

### 2.3 Artificial contamination of bread

Toast bread was seeded with 1 ml of SARS‐CoV‐2 at a final concentration of ~6 log TCID_50_/g. After that, the breads were subjected to 630 watt in microwave for two different times: 30 s and 1 min.

### 2.4 Chemical analysis of meat products and bread

Moisture, fat, and protein contents of the samples were determined by the AOAC methods (AOAC, 1990). Fat and protein contents were measured by Soxhlet and Kjeldahl methods, respectively. Moisture content was determined by weight loss in a drying oven (5 hr at 105℃). The pH values of homogenate samples were determined at ambient temperature using a pH meter (Metrohm, Switzerland).

### 2.5 Statistical analysis

All experiments and analysis were performed in triplicate, and the data were presented as means ±standard deviation.

## 3 RESULTS AND DISCUSSION

### 3.1 Effect of heat processing of meat products on SARS‐CoV‐2

As mentioned before, in this study, the effect of cooking and frying of sausages and hamburger on stability of SARS‐CoV‐2 was investigated. Fat, protein, and moisture contents and pH of tested meat products are shown in Table 1. Furthermore, the effect of heating process on the survival of SARS‐CoV‐2 in sausages and hamburger is illustrated in Table 2. According to Table 2, it can be figured out that heating process of hamburger and sausages led to inactivation of SARS‐CoV‐2. The result showed that frying of hamburger at 225ºC for about 6 and 10 min (3 and 5 min for each side, respectively) resulted in complete inactivation of SARS‐CoV‐2 with initial concentration of 5.70 log TCID_50_/g. Therefore, it seems that frying at 225ºC for 6 or 10 min was adequate to make a 5‐log decrease in SARS‐CoV‐2 load in hamburger. These results revealed that the common process of hamburger frying can be effective in inactivation of SARS‐CoV‐2. In addition, cooking of sausage at 78ºC for either 20 or 30 min was effective in inactivation of SARS‐CoV‐2. These findings indicated that regular cooking that exerts in order to produce heat‐processed sausages was able to inactivate SARS‐CoV‐2, and a 5‐log decrease in SARS‐CoV‐2 load was observed in sausages as a consequence of cooking process at 78ºC for either 20 or 30 min.

**TABLE 1:** The pH and protein, fat, and moisture contents of different food products contaminated with the virus

Tested products	Composition			
	pH	Protein content (%)	Fat content (%)	Moisture (%)
Bread	5.24 ± 0.08	‐	1.83 ± 0.15	35.40 ± 0.22
Sausage 40%	6.17 ± 0.02	9.45 ± 0.36	11.89 ± 0.77	52.33 ± 0.91
Sausage 80%	6.01 ± 0.03	14.74 ± 0.12	3.96 ± 0.28	66.20 ± 1.35
Hamburger	‐	12.77 ± 0.31	14.94 ± 0.15	61.55 ± 0.88

**TABLE 2:** The effects of various processing on the stability of SARS‐CoV‐2 in bread and meat products*

Product	Cooking (ºC)	Frying (ºC)	Microwave (watt)	Process time	Initial virus titer (log TCID_50_/mL)	Post process virus titer (log TCID_50_/mL)
Toast bread	‐	‐	630	30 s	5.70 ± 0.04	ND
Toast bread	‐	‐	630	1 min	5.70 ± 0.06	ND
Hamburger	‐	225	‐	3 min	5.70 ± 0.05	ND
Hamburger	‐	225	‐	5 min	5.70 ± 0.07	ND
Sausages (40%)	78	‐	‐	20 min	5.70 ± 0.06	ND
Sausages (40%)	78	‐	‐	30 min	5.70 ± 0.04	ND
Sausages (80%)	78	‐	‐	20 min	5.70 ± 0.07	ND
Sausages (80%)	78	‐	‐	30 min	5.70 ± 0.06	ND

Abbreviations: *TCID_50_, Fifty percent tissue culture infected dose; ND, not detected.

As far as we know, meat and meat products are good sources of proteins, essential lipids, some vitamins, and iron (Kovacevic et al., 2019; Kulczyński et al., 2019; Yousefi et al., 2018). However, they can be a source of deleterious food pathogens such as *Escherichia coli* O157: H7, *Listeria monocytogenes*, *Salmonella* spp., and hepatitis E virus (Birgen et al., 2020; Yekta et al., 2020; Yousefi et al., 2020). Therefore, meat and meat products can be considered as a potential source of outbreaks, especially in poor hygiene conditions. Accordingly, viral transmission of SARS‐CoV‐2 might be carried out through meat and meat products, particularly in the unhygienic processing conditions as well as cross‐contamination. It has been proposed that raw meat and meat products are exposed to proper heating process (more than 60ºC) for at least 30 min before consumption (Yekta et al., 2020). As mentioned previously, a 6 log decrease in SARS‐CoV‐2 load was observed as consequence of heat treatment of 70℃ for about 5 min and since, during the cooking process of meat products such as cooked sausages, the core temperature of these products reaches 74–76℃, it seems that this thermal process is sufficient to inactivate the SARS‐CoV‐2 (Chin & Poon, 2020; Sukumaran et al., 2018). Similarly, we found a complete inactivation of SARS‐CoV‐2 thorough cooking of sausages. These results indicate that there is no concern about the consumption of cooked meat products. However, it is necessary to be very careful in processing raw and precooked sausages in which no adequate heat treatment excreted. Furthermore, hamburger is one of the most popular meat products that is stored and sold frozen. Due to the fact that coronaviruses are highly stable in the freezing state and can even survive at −20℃ for about years (Han et al., 2020; World Health Organization., 2020), it is worth mentioning that SARS‐CoV‐2 viral load could not be decreased in contaminated hamburgers during freezing. Therefore, proper frying of hamburger is considered as the most important way to significantly inactivate SARS‐CoV‐2 in this product. It has been indicated that since the cold point in hamburger reaches the temperature of 72℃ during heat processing, destruction of SARS‐CoV‐2 viral load might occur (Lima Filho et al., 2019). Likewise, in this study, we found that proper frying of hamburger at 225℃ for at least 6 min resulted in destruction of SARS‐CoV‐2. Therefore, exerting adequate heating process would inactivate SARS‐CoV‐2, especially in foodstuff in which food composition such as sugar, protein, and fat affected survival of pathogens such as SARS‐CoV‐2. It has been indicated that at least a 4‐log reduction was occurred in coronavirus infectivity by applying thermal disinfection at 60, 65, and 80℃ for 30 min, 15 min, and 1 min, respectively (Kampf et al., 2020). It has been noted that thermal aggregation of the SARS‐CoV‐2 membrane protein is occurred through heat treatment and a complete denaturation of nucleocapsid protein of SARS‐CoV‐2 was observed at 55℃ for 10 min (Lee et al., 2005; Wang et al., 2004).

### 3.2 Effect of microwave processing on virus SARS‐CoV‐2 in bread

One of the main applications of microwave is reheating bread at home, so one of the objectives of this study was to investigate the effect of bread heating by microwave process on the stability of coronavirus. The composition of tested bread such as fat and moisture contents and pH is revealed in Table 1. Additionally, the survival of SARS‐CoV‐2 as a consequence of microwave process of toast bread is shown in Table 2. According to Table 2, it can be understood that exerting 630 watt for either 30 s or 1 min led to a 5‐log decrease in SARS‐CoV‐2 load in bread. These results indicated that using microwave power of 630 watt at least for 30 s in order to heat the bread resulted in inactivation of SARS‐CoV‐2.

Microwave is a form of electromagnetic waves with wavelengths of one meter to one millimeter and a frequency between 100 megahertz and 3,000 MHz. After passing through the medium, the absorbed microwave generates heat by vibrating and rubbing of the substance molecules for billions of times per second. The generated heat can exert antimicrobial effects (Ohtsu et al., 2011; Wang et al., 2020). It has been indicated that microwave at frequency of 2,450 and 915 MHz could be applied for pasteurization of ready‐to‐eat meals (Tang et al., 2018). Furthermore, it has been reported that microwave could be utilized as disinfectant agent for disinfection of hospital wastes and wastewater during COVID‐19 (Wang et al., 2020). In fact, during COVID‐19 pandemic, different recommendation and devices were proposed for sanitization of items. Microwave ovens due to their wide availability at home are considered as one of these strategies for sanitizing. However, it is required to establish a guideline for using microwave oven as sanitizer device, especially the ones include for elimination viruses from various food stuff (Aguilar‐Garib, 2020).

## 4 CONCLUSION

In this study, the effect of cooking and microwave processes on stability of SARS‐CoV‐2 in meat products and toast bread was investigated. The results revealed that frying of hamburger at 225℃ for about either 6 or 10 min resulted in a 5‐log decrease in SARS‐CoV‐2 load. Furthermore, cooking of sausage at 78℃ for at least 20 min could inactivate all the inoculated SARS‐CoV‐2 (5.70 log TCID_50_/g). These results indicated that exerting adequate heat treatment can be effective in elimination of the coronavirus that may enter into meat products during preparation, transportation, or retail. Additionally, this study showed that using microwave oven at power of 630 watt for at least 30 s can significantly inactivate the inoculated virus (5.70 log TCID_50_/g). This finding indicated that microwave oven can be feasible in removing SARS‐CoV‐2 from the bread that might be contaminated after baking in bakery or during distribution and sales in retail. However, in order to ensure about the effectiveness of microwave ovens in elimination of SARS‐CoV‐2, it is necessary to prepare as suitable guideline for the people to utilize not only about heating bread but also about other foods that are heated in the microwave. Generally, it could be stated that foodstuff might be contaminated with the SARS‐CoV‐2 and act as a carrier in the transmission of COVID‐19, and given that this cross‐contamination of food during preparation, distribution, and sales in retails is inevitable, it is necessary that the consumer be aware of the importance of proper heating treatment of food products as well as microwave ovens at right time and power in elimination of SARS‐CoV‐2.

## CONFLICT OF INTEREST

The authors declare no conflict of interest.

## ETHICAL APPROVAL

This study does not involve any human or animal testing.

## Data Availability

The data that support the findings of this study are available from the corresponding author upon reasonable request.
